# Long-term staged orthopedic reconstruction in a patient with sickle cell disease and complex multijoint fusion

**DOI:** 10.1093/jscr/rjag303

**Published:** 2026-04-28

**Authors:** Danil V Chernov, Nicholas Frappa, Matthew G Alben, Joshua Slowinski, Ryan Riley, Gabrielle Hartman, Jennifer Gurske-dePerio

**Affiliations:** Jacobs School of Medicine and Biomedical Sciences, Department of Orthopaedics, 955 Main Street, Buffalo, NY 14203, United States; Jacobs School of Medicine and Biomedical Sciences, Department of Orthopaedics, 955 Main Street, Buffalo, NY 14203, United States; University at Buffalo Department of Orthopaedics and Sports Medicine, 462 Grider Street, Buffalo, NY 14215, United States; University at Buffalo Department of Orthopaedics and Sports Medicine, 462 Grider Street, Buffalo, NY 14215, United States; University at Buffalo Department of Orthopaedics and Sports Medicine, 462 Grider Street, Buffalo, NY 14215, United States; University at Buffalo Department of Orthopaedics and Sports Medicine, 462 Grider Street, Buffalo, NY 14215, United States; University at Buffalo Department of Orthopaedics and Sports Medicine, 462 Grider Street, Buffalo, NY 14215, United States

**Keywords:** sickle cell disease, ankylosis, subtalar coalition, opioid dependence, long-term management

## Abstract

Sickle cell disease is associated with numerous musculoskeletal complications, but joint ankylosis and congenital skeletal fusion are rarely reported. We present the case of a young woman with sickle cell disease and complex multijoint pathology, including hip ankylosis of uncertain etiology and congenital hindfoot fusion. Beginning in adolescence, she underwent staged orthopedic reconstruction over more than a decade, including bilateral total hip arthroplasty, lower-extremity deformity correction, arthrodesis, treatment of prosthetic joint infection, management of recurrent soft-tissue ulceration, and multiple hardware removals. Her clinical course was complicated by impaired wound healing, infection risk, and chronic pain requiring multidisciplinary management. Despite these challenges, staged surgical intervention resulted in meaningful functional improvement and improved ambulatory capacity. This case highlights the feasibility of complex, longitudinal orthopedic reconstruction in patients with sickle cell disease while emphasizing the elevated risks of infection, wound complications, and pain management challenges.

## Introduction

Sickle cell disease (SCD) is an autosomal recessive hemoglobinopathy characterized by vaso-occlusive crises, chronic hemolytic anemia, and progressive multisystem complications [1]. Improvements in hematologic care have increased life expectancy, resulting in more patients with SCD presenting for complex orthopedic management [2]. Common musculoskeletal manifestations include osteonecrosis, osteomyelitis, septic arthritis, chronic ulceration, and structural deformities related to recurrent ischemia and bone remodeling [3, 4].

Joint ankylosis is rarely reported in patients with SCD and is typically described as an acquired sequela of ischemia or infection rather than a congenital abnormality [5, 6]. Congenital tarsal coalition, resulting from incomplete segmentation of primitive mesenchyme, is itself uncommon and has not been previously reported in association with SCD [7, 8]. We present a case of complex multijoint pathology involving hip ankylosis of uncertain etiology and congenital hindfoot fusion in a patient with SCD who underwent staged orthopedic reconstruction over more than a decade, highlighting the challenges and considerations inherent to long-term surgical management in this population.

## Case report

**Figure 1:**
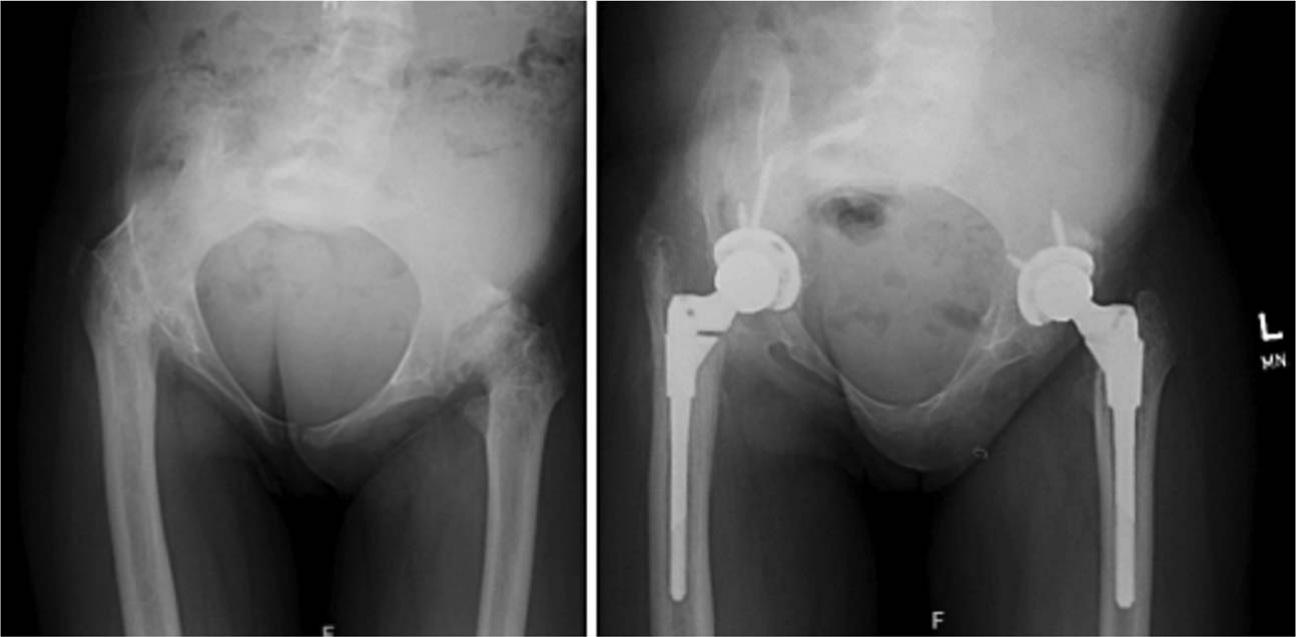
Anteroposterior radiographs of the pelvis demonstrating bilateral hip ankylosis prior to surgical intervention (left) and following staged bilateral total hip arthroplasty with restoration of joint alignment (right).

A 25-year-old African American woman with SCD presented with bilateral hip fusion, congenital fusion of the left subtalar joint, and long-standing contractures of the left knee and ankle. Her early medical history was notable for a sickle cell crisis in childhood while living in Africa, during which she was treated nonoperatively with casting for presumed hip pathology. No operative records were available from this period, and there was no history of surgery involving the left foot or ankle. Subsequent radiographs obtained after immigration demonstrated congenital fusion of the left ankle and subtalar joints in a fixed plantarflexed position (Fig. 1). The contralateral right foot and ankle were unaffected.

**Figure 2:**
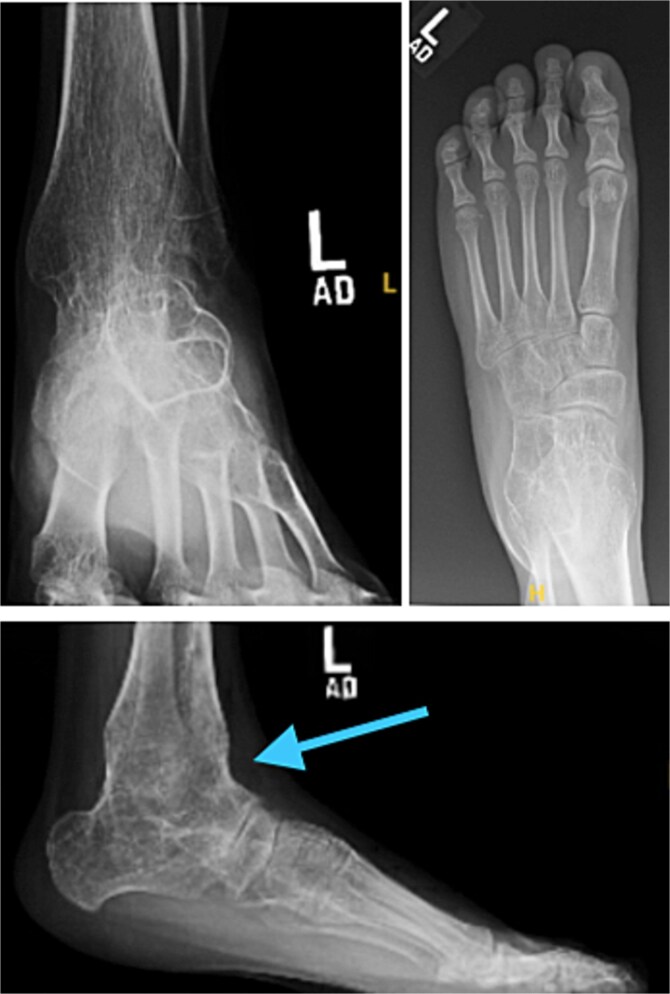
Preoperative radiographs of the left foot and ankle demonstrating congenital subtalar coalition with associated rigid equinus deformity. Anteroposterior and oblique views show hindfoot osseous fusion and altered midfoot alignment, while the lateral view (arrow) highlights the fixed plantarflexed position of the ankle and hindfoot.

In December 2015, imaging in the USA revealed fusion of the right hip and severe dysplasia of the left hip. She underwent staged bilateral total hip arthroplasty, including surgical release of the fused right hip (Fig. 2). Postoperatively, she developed a prosthetic joint infection of the right hip with *Salmonella* species. Management consisted of irrigation and debridement with modular component exchange, followed by targeted antimicrobial therapy, resulting in successful infection control and implant retention.

**Figure 3:**
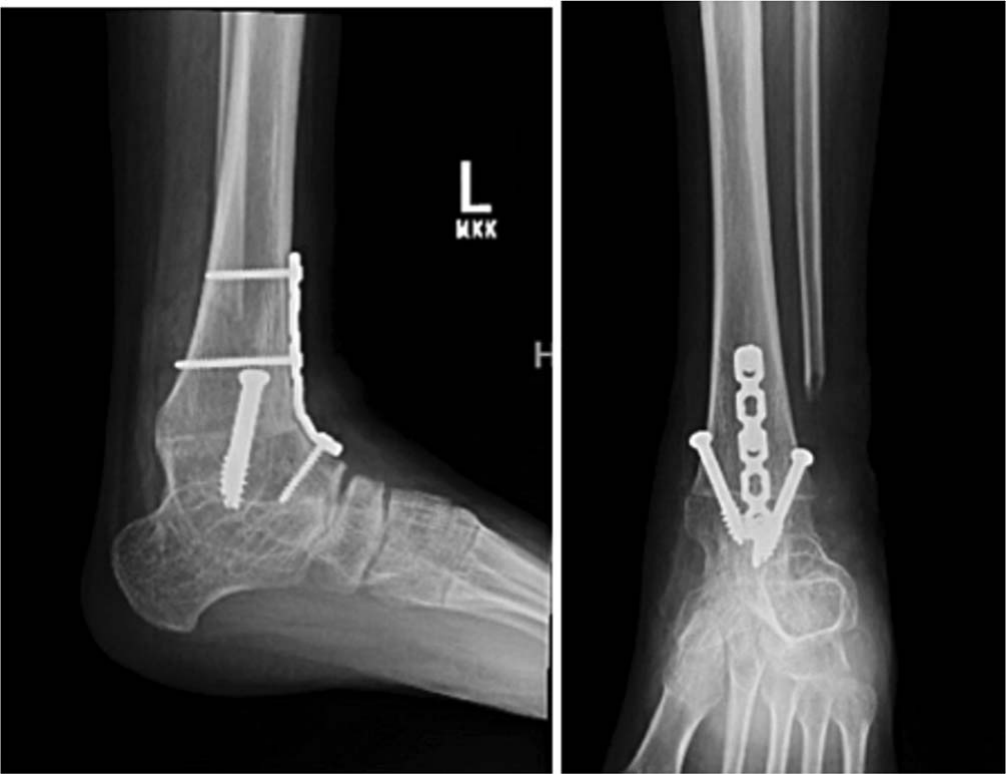
Postoperative radiographs of the left ankle and hindfoot following reconstructive surgery for rigid equinus deformity associated with subtalar coalition. The lateral and anteroposterior views demonstrate distal tibial and fibular osteotomies with internal fixation and improved hindfoot alignment.

In December 2016, she underwent left ankle and hindfoot reconstruction for rigid equinus deformity related to a congenital subtalar coalition (Fig. 3). The procedures included distal tibial and fibular osteotomies, Achilles tendon lengthening, and soft tissue releases. The subtalar joint was confirmed to be congenitally fused, with arthritic changes at the talonavicular joint. Talonavicular arthrodesis was deferred. Postoperatively, alignment and hindfoot stability improved.

In February 2018, a recurrent full-thickness ulcer developed over the left lateral ankle. Surgical debridement revealed devitalized soft tissue without bone or hardware exposure. Negative-pressure wound therapy and antimicrobial treatment resulted in healing.

**Figure 4:**
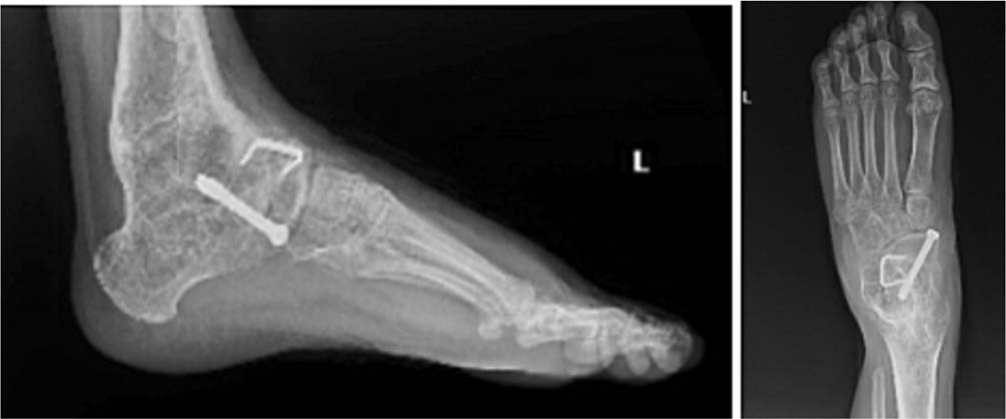
Radiographs of the left foot demonstrating progressive talonavicular joint degeneration and dorsal exostosis formation. Lateral and dorsoplantar views show degenerative changes and bony overgrowth contributing to midfoot pain and mechanical symptoms prior to subsequent surgical intervention.

In April 2020, the patient presented with localized pain and hardware-related symptoms involving the left foot and ankle. Imaging demonstrated prominent dorsal exostoses of the talus and navicular with progressive talonavicular joint degeneration (Fig. 4). She underwent removal of prior tibial and talar hardware with exostectomy of the dorsal talus and navicular, resulting in symptomatic improvement.

**Figure 5:**
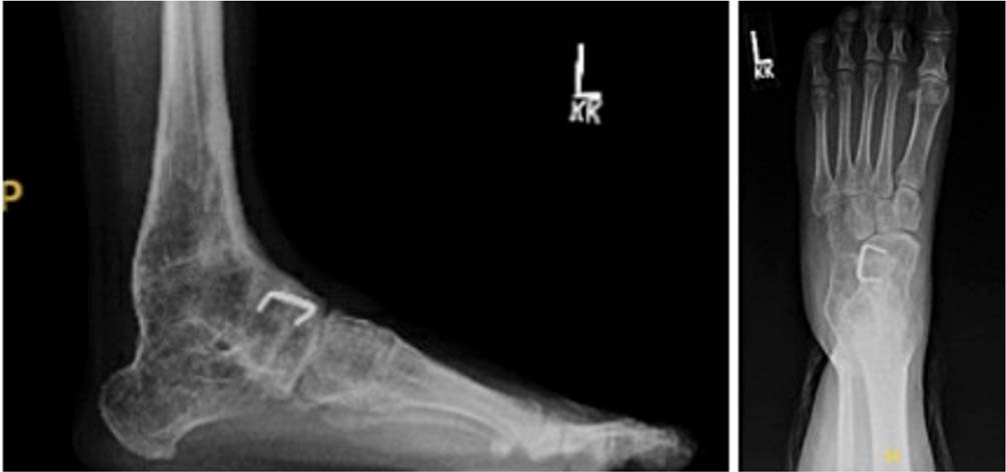
Postoperative radiographs of the right ankle following hardware removal and anterior tibial exostectomy. The lateral and dorsoplantar views demonstrate removal of prior fixation with maintained alignment and evidence of a healed ankle fusion.

Between 2020 and 2025, the patient underwent multiple additional procedures for progressive midfoot degeneration, nonunion, symptomatic exostoses, and hardware irritation, including talonavicular arthrodesis with autologous bone grafting and staged hardware removals. In August 2025, she underwent right ankle hardware removal and anterior tibial exostectomy, with confirmation of healed fusion and postoperative symptomatic improvement (Fig. 5).

## Discussion

SCD is associated with a broad spectrum of musculoskeletal complications, most commonly avascular necrosis, chronic osteomyelitis, septic arthritis, and nonhealing ulcers resulting from recurrent vaso-occlusive injury and impaired bone perfusion [3, 9]. Congenital joint ankylosis has been rarely reported in patients with SCD. In this case, the etiology of the patient’s hip fusion could not be definitively established and may represent a combination of early-life sickle cell–related pathology, prior immobilization, and infection. In contrast, the subtalar coalition demonstrated imaging features consistent with congenital fusion, a condition that is itself uncommon and has not been previously reported in association with SCD.

The surgical management of this patient required a staged approach tailored to both the severity of her deformities and the systemic risks associated with SCD. Bilateral total hip arthroplasty was prioritized to restore sitting balance and ambulation, despite the added complexity of releasing a fused hip. Prosthetic joint infection remains a well-recognized complication in patients with SCD, particularly with *Salmonella* species, due to functional asplenia and impaired immune response [10, 11]. In this case, early identification of infection and retention of well-fixed components through irrigation, debridement, and modular exchange allowed for successful eradication of infection while preserving the reconstruction.

Impaired wound healing represented a recurrent challenge throughout this patient’s course. Chronic ulceration in SCD is multifactorial, arising from microvascular occlusion, chronic anemia, endothelial dysfunction, and local tissue hypoxia [12]. Surgical intervention in previously infected or scarred tissue further increases the risk of delayed healing and breakdown [12]. Successful management requires prompt surgical debridement, negative-pressure wound therapy, and close collaboration with infectious disease specialists.

Pain management was a critical component of this patient’s care and significantly influenced the perioperative planning. Patients with SCD frequently experience chronic pain and are at increased risk for long-term opioid use, which can complicate postoperative recovery and functional outcomes [13, 14]. In this case, a history of opioid dependence and complex regional pain syndrome necessitated an individualized, multimodal analgesic approach incorporating regional anesthesia, nonopioid adjuncts, and careful opioid titration.

The patient continues to receive routine hematologic care, including hydroxyurea therapy and intermittent blood transfusions, which play an important role in reducing vaso-occlusive episodes that may jeopardize orthopedic reconstructions. At long-term follow-up, staged surgical intervention resulted in meaningful functional improvement, including improved sitting balance, plantigrade weight bearing, and independent ambulation with assistive devices. Despite these gains, her course was marked by recurrent soft-tissue complications and the need for additional procedures related to reactive bone formation and hardware irritation. This case illustrates both the potential benefits and persistent challenges of complex orthopedic reconstruction in patients with sickle cell disease and underscores the importance of sustained interdisciplinary follow-up.

## Conclusion

Orthopedic reconstruction in patients with SCD and complex joint pathology is feasible but carries elevated risks of infection, impaired wound healing, and prolonged pain management challenges. Successful outcomes require individualized surgical planning, heightened perioperative vigilance, and close collaboration among orthopedic, hematologic, infectious disease, and pain management teams. This case highlights the importance of long-term follow-up and multidisciplinary care when treating rare and complex musculoskeletal presentations in patients with SCD.
